# Assessment of obstructive sleep apnea rate and associated factors among Lebanese adults: a cross-sectional study

**DOI:** 10.3389/fpubh.2025.1443920

**Published:** 2025-02-13

**Authors:** Tala Kaddoura, Mohamad Hodroj, Bilal Chmeis, Fatima Rammal, Sandrella Bou Malhab, Sara Mansour, Amal Akour, Sami El Khatib, Basile Hosseini, Souheil Hallit, Diana Malaeb, Hassan Hosseini

**Affiliations:** ^1^School of Pharmacy, Lebanese International University, Beirut, Lebanon; ^2^Faculty of Medicine, American University of Beirut, Beirut, Lebanon; ^3^Institut National de Santé Publique d’Épidémiologie Clinique et de Toxicologie-Liban (INSPECT-LB), Beirut, Lebanon; ^4^Department of Pharmacology and Therapeutics, College of Medicine and Health Sciences, United Arab Emirates University, Al-Ain, United Arab Emirates; ^5^Department of Biopharmaceutics and Clinical Pharmacy, School of Pharmacy, The University of Jordan, Amman, Jordan; ^6^Department of Biomedical Sciences, Lebanese International University, Bekaa, Lebanon; ^7^Center for Applied Mathematics and Bioinformatics (CAMB), Gulf University for Science and Technology, Mubarak Al-Abdullah, Kuwait; ^8^Hospices Civils de Lyon, Lyon, France; ^9^School of Medicine and Medical Sciences, Holy Spirit University of Kaslik, Jounieh, Lebanon; ^10^Department of Psychology, College of Humanities, Effat University, Jeddah, Saudi Arabia; ^11^Applied Science Research Center, Applied Science Private University, Amman, Jordan; ^12^College of Pharmacy, Gulf Medical University, Ajman, United Arab Emirates; ^13^UPEC-University Paris-Est, Creteil, France; ^14^RAMSAY SANTÉ, HPPE, Champigny sur Marne, France

**Keywords:** obstructive sleep apnea, hypertension, sex, correlates, COVID-19

## Abstract

**Objectives:**

To estimate the rate of obstructive sleep apnea (OSA) and associated factors among Lebanese adults.

**Methods:**

A cross-sectional study was conducted in February–April 2022; 1,202 Lebanese adults were enrolled. The sample was collected among several Lebanese governorates through an anonymous online self-reported questionnaire. The STOP-BANG questionnaire was used as a screening tool to estimate the OSA risk. “Low OSA risk” is established with 0–2 positive answers, “Intermediate OSA risk” is established by 3–4 positive answers while “High OSA risk” is established by 5–8 positive answers.

**Results:**

This study showed that 743 (62.4%) of the sample had low risk for OSA, 357 (30.0%) had moderate, and 90 (7.6%) had high risk for OSA. Older age (aOR = 1.05) and having hypertension (aOR = 7.80) were associated with higher odds of moderate OSA. Female sex (aOR = 0.04) was significantly associated with lower odds of moderate OSA compared to males. Older age (OR = 1.17), higher BMI (OR = 1.14), hypertension (OR = 18.55), and having severe COVID-19 infection compared to mild (OR = 4.30) were significantly associated with higher odds of high OSA, whereas female sex (OR = 0.002) and being married compared to single (aOR = 0.23) were associated with lower odds of high OSA.

**Conclusion:**

This study showed that most Lebanese adults have low risk for OSA. It also confirmed that sex, age, obesity, hypertension, and severe COVID-19 were associated with high OSA risk. These associated factors call for future research exploring the causes including the potential effect of social, economic, and political instability, local customs, and environmental factors. Moreover, future health campaigns should be assigned to increase awareness among Lebanese population regarding the prevention of OSA through lifestyle modifications.

## Introduction

Obstructive sleep apnea (OSA) is a sleep disorder characterized by recurrent episodes of complete (apnea) or partial (hypopnea) collapse of the upper airway which impairs regular ventilation during sleep. OSA increases the risk of metabolic, cardiovascular and neurocognitive disorders due to hypoxemia and oxidative stress (1). OSA accounts for more than 85% of sleeping disorders. It can be asymptomatic in majority of those having moderate to severe OSA (2). Estimates of the prevalence of OSA vary based on the diagnostic criteria used, a recent study showed that the estimated prevalence ranges from 9 to 38% in community-screened populations (3). According to the American Academy of Sleep Medicine (AASM), undiagnosed sleep apnea costs $27 billion annually linked to the comorbid health risks (4). Several risk factors contribute to OSA occurrence, which are divided into unmodifiable and modifiable factors. Unmodifiable risk factors include sex, age, and genetic predisposition, while modifiable ones include obesity, specific medication utilization, hypothyroidism disorders, smoking, and nasal congestion (5). According to previous studies, men demonstrate higher risk for developing sleep apnea and higher severity of the disease than women (6). In addition, patients older than 60 years and obese with BMI > 30 kg/m^2^ had higher prevalence of OSA (7, 8). Furthermore, OSA is highly interrelated with different morbidities such as diabetes mellitus (DM) (9), progressive renal function decline (10), depression (11, 12), as well as cardiovascular disorders such as: hypertension, heart failure, atrial fibrillation, stroke, and coronary artery disease (13). Indeed, cardiovascular diseases existence is highly interrelated and bidirectionally associated with OSA providing a strong causal and resultant association. It can be also correlated with poor pregnancy outcomes including postpartum hemorrhage, premature delivery, congenital abnormalities (14), in addition to other disorders like gastro-esophageal reflex disorder (GERD) (15). Not only physiological changes occur, but also patients will become vulnerable to excessive daytime drowsiness, inattention, and irritability which will increase their risk of falling asleep while doing their basic daily activities, putting them at a high risk of all-cause mortality and work-related accidents (16). According to a survey done in Lebanon, early AM hours fatal crashes were associated with the drivers’ fatigue and sleepiness (17). Afterwards, systematic and meta-analytic reviews provided robust evidence that OSA plays a critical role in the development of broad-spectrum cognitive dysfunctions such as attention and vigilance deficits, verbal and visual delayed long-term memory, and executive function decline (18). Lebanese patients today need to be aware of the common pathologies and symptoms that should lead them to consult a physician for early diagnosis of OSA. A research study done in Lebanon has shown that patients could be underdiagnosed to due asymptomatic presentation in most of the cases (19). Another study conducted in Beirut showed that although 31% participants were at high risk for sleep apnea, only 5% were diagnosed by a physician (20). Chronic stress from political, social and economic instability lead to high rates of smoking (21), and unhealthy dietary habits (22) which contribute to key OSA risk factors like obesity and airway inflammation. Therefore, the aim of this study is to thoroughly evaluate the risk of OSA in a large, sample of Lebanese adults, with a particular focus on how various health factors including comorbidities, lifestyle choices, and demographic characteristics contribute to the likelihood of developing OSA. By examining these factors in a comprehensive manner, this study seeks to identify key determinants that may influence OSA risk within the Lebanese population and provide a more clear understanding of its prevalence and contributing factors.

## Materials and methods

### Study design

This cross-sectional study was conducted between February and April 2022 after the lockdown period imposed by the government for the coronavirus disease 2019 (COVID-19) pandemic. We used an anonymous self-administered questionnaire developed on Google® Forms and posted on several social media platforms. The link was shared among participants using the social media and sent to people from all districts/governorates of Lebanon (Beirut, Mount Lebanon, North Lebanon/Akkar, South Lebanon/Nabatieh, and Bekaa/Hermel-Baalbeck) using the snowball technique. Participants were asked to fill the survey online which takes between 15 and 20 min to be complete and send the link to other participants too, which explains the snowball sampling technique used.

### Inclusion and exclusion criteria

Eligibility criteria was mentioned in the first section of the survey that clearly specified the exclusion criteria. Excluded were, any participant younger than 18 years and patients with cognitive impairment or mental disorder. All participants knew the purpose of the study and gave their informed consent before starting the questionnaire. Participants received no compensation for their participation, which was completely voluntary.

### Sample size calculation

We used G*Power software was used to determine the sample size. The minimum required sample size was 600 participants, considering an alpha error of 5%, a power of 95%, a minimal model r-square of 5% and allowing 20 predictors to be included in the model.

### Tools and procedure

The questionnaire is divided into three sections. The first part included: socio-demographic characteristics including age, sex, weight, height, marital status, employment status, work type, monthly income, residency area (Beirut, Mount Lebanon, South, North, Akkar, Baalbek-Hermel, and Nabatieh), and household crowding index (calculated by taking the total number of co-residents per household excluding the newborn infant divided by the total number of rooms excluding the kitchen and bathrooms). Furthermore, it encompassed data about the social history for smoking and alcohol consumption. This section included also an assessment of health-related disorders including excessive drowsiness per day, waking up gasping and choking, dry mouth or sore throat after waking up, morning headaches, lack of concentration, mood swings, depression, forgetfulness, and swelling in the legs. The second part incorporated personal diseases and medication intake documented through assessing previous diagnosis of OSA, stroke, heart failure, diabetes mellitus, hypertension, and other chronic non-communicable diseases. Also, COVID 19 retraction history was documented, including symptoms, vaccination status, and the vaccine status.

The third part included the different scales used:

Patient Health Questionnaire (PHQ-9): an Arabic version validated scale in Lebanon (23) to retrieve information to screen for the presence and severity of depression. The questionnaire is composed of 9 multiple choice questions rated from 0 = not at all to 3 = nearly every day (24). Total score is calculated by summing all the choices together (24).
Lebanese Anxiety Scale (LAS-10) is a validated tool utilized to screen for anxiety in adults (25) and adolescents (26). It is composed of 10 self-reported items, the total score is calculated by summing all the answers, with higher scores reflecting that the participant is more anxious (25). Answers were scaled from 0 to 4 for each question with: 0 not present, 1 mild, 2 moderate, 3 severe, 4 very severe. The Cronbach’s alpha is 0.947.
Beirut Distress Scale (BDS): It is a valid and reliable tool to screen for psychological distress, influencing mood, and physical and cognitive functions among the Lebanese population (27). It is composed of 10 items each scored from 0 = never to 3 = always. The Cronbach’s alpha is 0.097.
STOP-BANG questionnaire: It is used to screen for OSA, which is a reliable, concise, an easy-to-use screening tool for obstructive sleep apnea. This questionnaire is valid in Arabic and reported snoring behavior, tiredness, gasping, hypertension, and neck circumference (28). It consists of eight dichotomous (yes: positive answer/no: negative answer) items related to the clinical features of sleep apnea. The total score ranges from 0 to 8. Patients can be classified for OSA risk based on their respective scores. “Low OSA risk” is established with 0–2 positive answers, “Intermediate OSA risk” is established by 3–4 positive answers while “High OSA risk” is established by 5–8 positive answers. Or a minimum of 2 on the STOP questions in addition to male sex or BMI > 35 kg/m^2^ or an elevated neck circumference (>43 cm in males or > 41 cm in females) (29). The Cronbach’s alpha is 0.7 (28).

### Statistical analysis

Data was analyzed on the Statistical Package for Social Sciences version 25. Descriptive statistics was performed through the use of mean and standard deviation for continuous measures, counts, and percentages for categorical variables. Cronbach’s alpha values were recorded for the reliability of the analysis of all scales. Association between multiple categorical variables and OSA risk was evaluated using a Chi-square test in the bivariate analysis, whereas the ANOVA test was used to compare multiple means in the case of continuous variables. Bonferroni correction was applied for multiple testing by dividing 0.05 (5% risk of error) by the total number of variables included in the analysis (=22). The corrected *p*-value for significance was estimated at 0.05/22 = 0.002. Multinomial logistic regression was performed taking the obstructive sleep apnea risk (OSA risk) as the dependent variable. Factors that showed a significant p-value in the bivariate analysis were used as independent variables in the final model. Statistical significance was considered at *p* ≤ 0.05 in the final model.

## Results

## Socio-demographic characteristics

A total number of 1,190 persons was enrolled in the study, with a mean age of 30.66 years (±12.56). Females represented 64.7% of the sample. We found that 743 (62.4%) of the study population had low risk for OSA, 357 (30.0%) had moderate risk, and 90 (7.6%) had high risk for OSA. Other characteristics of the sample can be found in Table 1.

**Table 1:** Socio-demographic characteristics of the study participants.

Variables	Frequency (percentage) n (%)
Sex	
Male	416 (35.0)
Female	774 (65.0)
Marital status	
Single/divorced/widowed	786 (66.1)
Married	404 (33.9)
Employment status	
Unemployed/retired	553 (46.6)
Employed	634 (53.4)
Dwelling region	
Beirut	363 (30.5)
Mount Lebanon	413 (34.7)
South Lebanon/Nabatieh	350 (29.4)
North Lebanon/Akkar	45 (3.8)
Bekaa/Baalbeck-Hermel	19 (1.6)
Residency area	
Urban	704 (59.2)
Rural	485 (40.8)
Social history	
Smoking	511 (43.0)
Alcohol	133 (11.2)
Symptoms	
Day sleep	333 (28.0)
Excessive drowsiness	225 (18.9)
Waking up gasping	50 (4.2)
Snoring loudly	169 (14)
Dry mouth at waking up	219 (18.4)
Morning headache	208 (17.5)
Lack of concentration	214 (18.0)
Mood swings	414 (34.8)
Forgetfulness	272 (22.9)
Swelling of the legs	40 (3.4)
Past medical history	
Depression	180 (15.1)
Congestive heart failure	9 (0.8)
Diabetes mellitus type 1	27 (2.3)
Diabetes mellitus type 2	27 (2.3)
Hypertension	100 (8.4)
Parkinson	3 (0.3)
Lung diseases	78 (6.5)
PCOS	75 (6.3)
Irritability/Anxiety	166 (13.9)
Use of sedatives	78 (6.6%)
COVID history	
Previous COVID-19 infection	692 (58.3%)
Hospitalized due to COVID-19 infection	22 (2.2%)
COVID-19 vaccine intake	943 (79.3%)
	Mean (±SD)
Age (in years)	30.66 (12.56)
Body mass index (kg/m^2^)	24.69 (5.06)
Household crowding index (person/room)	1.04 (0.58)
Sleeping hours	7.26 (1.47)
Neck circumference	41.18 (3.91)

### Bivariate analysis associated with OSA risk

The bivariate analysis results showed that a higher risk for obstructive sleep apnea was significantly found in males vs. females, in married persons compared to single ones, being employed, smokers compared to nonsmokers, those who drink alcohol vs. not, and in participants who have congestive heart failure, diabetes mellitus type 2, hypertension, lung diseases, hormonal disorders, and narrowed airways compared to not. Moreover, higher age, BMI, depression, anxiety and stress means were found in the group at high risk of OSA compared to the low and intermediate risk groups (Table 2).

**Table 2:** Bivariate analysis of the factors associated with OSA risk.

Variables	OSA risk			*p*-value
	Low risk N (%)	Moderate risk N (%)	High risk N (%)	
Sex				
Male	68 (16.3)	273 (65.6)	75 (18.0)	**<0.001**
Female	675 (87.2)	84 (10.9)	15 (1.9)	
Marital status				
Single	509 (64.8)	241 (30.7)	36 (4.6)	**<0.001**
Married	234 (57.9)	116 (28.7)	54 (13.4)	
Employment status				
Unemployed	375 (67.8)	148 (26.8)	30 (5.4)	**0.001**
Employed	367 (57.9)	207 (32.6)	60 (9.5)	
Bedroom sharing				
No	232 (58.6)	138 (34.8)	26 (6.6)	0.038
Yes	485 (64.2)	209 (27.7)	61 (8.1)	
Smoking				
Yes	276 (54)	183 (35.8)	52 (10.2)	**<0.001**
No	466 (68.9)	174 (25.7)	36 (5.3)	
Alcohol consumption				
Yes	53 (39.8)	67 (50.4)	13 (9.8)	**<0.001**
No	690 (65.4)	289 (27.4)	76 (7.2)	
Swelling of the legs				
Yes	20 (50)	14 (35)	6 (15)	0.113
No	721 (62.8)	343 (29.9)	84 (7.3)	
Congestive heart failure				
Yes	1 (11.1)	3 (33.33)	5 (55.6)	**<0.001**
No	742 (62.8)	354 (30.0)	85 (7.2)	
Diabetes mellitus type 1				
Yes	13 (48.1)	10 (37.0)	4 (14.8)	0.194
No	730 (62.8)	347 (29.8)	86 (7.4)	
Diabetes mellitus type 2				
Yes	9 (33.3)	7 (25.9)	11 (40.7)	**<0.001**
No	734 (63.1)	350 (30.1)	79 (6.8)	
Hypertension				
Yes	10 (10)	45 (45)	45 (45)	**<0.001**
No	733 (67.2)	312 (28.6)	45 (4.1)	
Parkinson				
Yes	1 (33.3)	1 (33.3)	1 (33.3)	0.212
No	741 (62.5)	356 (30)	88 (7.4)	
Lung diseases				
Yes	39 (51.3)	25 (32.9)	12 (15.8)	0.011
No	704 (63.2)	332 (29.8)	78 (7.0)	
Hormone disorder				
Yes	63 (80.8)	13 (16.7)	2 (2.6)	**0.002**
No	679 (61 s.2)	343 (30.9)	88 (7.9)	
Nasal congestion				
Yes	76 (60.3)	35 (27.8)	15 (11.9)	0.148
No	666 (62.7)	321 (30.2)	75 (7.1)	
Narrowed airways				
Yes	22 (46.8)	17 (36.2)	8 (17.0)	0.016
No	720 (63.2)	338 (29.6)	82 (7.2)	
Previous COVID-19 infection				
Yes	446 (64.5)	194 (28.0)	52 (7.5)	0.228
No	296 (59.8)	161 (32.5)	38 (7.7)	
Hospitalized due to COVID-19 infection				
Yes	10 (45.5)	7 (31.8)	5 (22.7)	0.009
No	621 (64.8%)	274 (28.6%)	63 (6.6)	
COVID-19 symptoms severity				
Mild	229 (65.1%)	106 (30.1%)	17 (4.8%)	**0.001**
Moderate	180 (65.5%)	75 (27.3%)	20 (7.3%)	
Severe	39 (62.9%)	11 (17.7%)	12 (19.4%)	
COVID-19 vaccine intake				
Yes	596 (63.2%)	273 (29.0%)	74 (7.8%)	0.261
No	146 (59.3%)	84 (34.1%)	16 (6.5%)	
Age	27.44 ± 8.62	33.28 ± 13.99	46.86 ± 18.08	**<0.001**
Body mass index	23.42 ± 4.53	26.20 ± 4.73	29.29 ± 6.17	**<0.001**
Household crowding index	1.05 ± 0.56	0.98 ± 0.52	1.19 ± 0.84	0.007
Depression	6.53 ± 4.82	5.48 ± 4.71	6.91 ± 4.68	**0.001**
Anxiety	14.26 ± 11.64	11.83 ± 10.97	16.31 ± 10.69	**<0.001**
Stress	9.29 ± 6.54	7.93 ± 6.12	9.59 ± 7.04	0.003

Numbers in bold indicate significant *p* values (*p* ≤ 0.002) after applying the Bonferroni correction for multiple analysis.

### Multinomial logistic regression: OSA risk is the dependent variable

A multinomial logistic regression, taking moderate vs. low OSA risk as the dependent variable, showed that older age (aOR = 1.05) and having hypertension vs. not (aOR = 7.80) were associated with higher odds of having moderate OSA. Female sex (aOR = 0.04) was significantly associated with lower odds of having moderate OSA compared to males (Table 3, Model 1).

**Table 3:** Multinomial logistic regression taking OSA risk as the dependent variable (low OSA risk [STOP-BANG score 0–2] taken as the reference category).

Model 1: moderate OSA risk [STOP-BANG score 3–4]			
Factor	aOR	95% CI	*p*
Age	1.05	1.15; 1.08	0.005
Sex (females vs. males*)	0.04	0.02; 0.07	<0.001
Hypertension (yes vs. no*)	7.80	2.29; 26.58	0.001
Model 2: high OSA Risk [STOP-BANG score > 4]			
Age	1.17	1.11; 1.23	<0.001
Body mass index	1.14	1.03; 1.25	0.009
Sex (females vs. males*)	0.002	0.001; 0.01	<0.001
Marital status (married vs. single*)	0.23	0.07; 0.73	0.013
Hypertension (yes vs. no*)	18.55	4.11; 83.78	<0.001
COVID-19 infection (severe vs. mild*)	4.30	1.05; 17.64	0.043

*Reference group; aOR = adjusted odds ratio; CI = confidence interval; Variables entered in the analysis were: age, sex, body mass index, depression, anxiety, employment status, marital status, smoking, alcohol consumption, hypertension, diabetes mellitus, congestive heart failure, hormone disorder, severity of COVID-19 infection (only significant results are included in the table).

A multinomial logistic regression, taking high vs. low OSA risk as the dependent variable, showed that older age (OR = 1.17), higher BMI (OR = 1.14), having hypertension vs. not (OR = 18.55), and having severe COVID-19 infection compared to mild (OR = 4.30) were significantly associated with higher odds of having high OSA, whereas female sex (OR = 0.002) and being married compared to single (aOR = 0.23) were significantly associated with lower odds of having high OSA (Table 3, Model 2).

## Discussion

To the best of our knowledge, this is the first large-scale study that assessed the correlates of obstructive sleep apnea risk among the Lebanese population. The results showed that older age, higher BMI, male sex, hypertension, marital status, and severe COVID-19 infection were associated with higher odds of having high OSA risk.

### Age and OSA risk

Our study showed that OSA is higher among patients with advanced age consistent with the findings from a previous study (3). A similar finding was observed in a study conducted on Saudi individuals, where the prevalence of OSA increased with age, independent of other potential confounding factors (30). Our results can be explained by the fact that older individuals suffer from reduced tethering of the upper airway due to the loss of the elastic recoil effect produced by the lung (1). In addition, older individuals suffer from an easier collapse in the airways caused by a reduction in the collagen, negative pressure reflux, and arousal threshold (1). Thus, the deposition of the parapharyngeal fat will increase, the soft palate will widen, and the bony shape seen around the pharynx will change. Moreover, the efficiency of the upper dilator muscles will fall with age accompanied by an incline in several health morbidities that act as risk factors for OSA occurrence (31, 32). Furthermore, our findings can be interpreted by the fact that hypertension is more prevalent in advanced age which helps in the understanding why OSA is more encountered in the older adult population (33).

### Obesity and OSA risk

Our study findings showed that OSA risk is higher at a BMI level of ≥35 kg/m2 consistent with findings from a previous study (30, 34). The association between obesity and OSA risk is a linear relationship that can be explained that in obesity more pressure will be applied on the airway tract through the increased disposition of aft around the neck area impairing the normal airflow and obstructing the respiratory tract site (35).

It has been documented that a 10% increase in body weight results in a sixfold increase in moderate to severe OSA and increases the apnea-hypopnea index (AHI) by 32% (36). Furthermore, it can be explained that obesity is not only a direct factor triggering OSA, it also acts as a confounding factor promoting other health morbidities such as hypertension, diabetes increased insulin resistance, and coronary artery diseases, which in return acts as major risk factors for OSA occurrence (37).

### Sex and OSA risk

Our findings confirmed that male sex is at a higher risk for developing OSA, which is consistence with the literature (3, 38–40). Our findings are consistent with those of Al Qattan et al., who evaluated the risk of OSA among Kuwaiti individuals (5). Our results can be interpreted by the fact that males have bigger lung volume and tends to gain weight more centrally than do females which probably results in men having more fat stored in the upper airway structures and abdomen sites than do women (41). Several studies have confirmed that men have larger airways than do women which confirms the propensity in the airway collapse in men more than women (1). It has been also highlighted that in the STOP-BANG questionnaire males receive an extra score placing them at a higher risk for OSA development (42). It is explained in the literature that men have more bulky tongues and soft palate and more fat deposition at the mandible site increasing the risk of developing OSA (41). In-addition to the unique anatomy and physiology pertaining to males, there are certain social and lifestyle factors which are more common in males that increase risk of OSA as smoking, alcohol consumption, and early work hour with diminished night sleep hours (43). There are different resources that justified the higher prevalence of OSA among males compared to females by elucidating the fact that females do not report symptoms of snoring and the reluctance of women to acknowledge symptoms of OSA and seek medical help (44).

### Hypertension and OSA risk

Our study reported a strong association between hypertension and a higher risk of OSA consistent with the findings by Borsini et al. and Silva et al. (45, 46). A study conducted in Turkey by Ozdemir et al. confirmed that the prevalence of OSA was as high as 9 times in hypertension compared to hypertension-free patients (32). The underlying reason can be explained by the fact that fluids at night are redistributed from the lower limbs to the neck imposes more airway pressure, exacerbates obstruction, and increases levels of angiotensin II and aldosterone in the nasopharynx and upper airway tissues in hypertension patients triggers OSA (47). In this study, OSA and hypertension coexisted together so it is difficult to build a unidirectional relationship since both are known to contribute to each other.

### Marital status and OSA risk

Our results showed that OSA risk is lower among married compared to single status consistent with the findings from another study (48). In contrast to our results, an Emirati study found higher prevalence of OSA risk in married groups compared to single, divorced or widowed groups (49). Our findings can be explained by the fact that insomnia-related symptoms mainly difficulty falling asleep is higher among unmarried individuals which increases the risk of developing OSA (50). It has been documented that irregular sleep rhythm and stronger night and day symptoms related to sleep disorders are more common among unmarried individuals which increases the risk of developing OSA (51, 52).

### Severity of COVID-19 symptoms and OSA risk

According to our findings, severe COVID-19 infection was associated with a higher risk of OSA consistent with the findings in another study conducted among US adults (53). Our findings can be explained by the fact that COVID-19 triggers an inflammatory state, especially in the lungs which can cause concentric fibrosis and can narrow the airway (54). It has been documented that patients infected with COVID-19 manifest with hypoxia and immune dysfunction that increases the risk to develop OSA (55).

### Depression, anxiety and stress and OSA risk

Our study identified a significant correlation between obstructive sleep apnea (OSA) and both depression and anxiety, while no such correlation was observed with stress. This was demonstrated through analyses using the PHQ-9 Depression Scale, the LAS Anxiety Scale, and the BDS Distress Scale. Starting with depression, our findings align with those of Lee et al., demonstrating that a high PHQ-9 score is significantly associated with a risk of OSA (56). Depression can be considered a risk factor for OSA, possibly due to the effects of OSA on insular neurons, which may experience damage or dysfunction (57). Sleep fragmentation and intermittent hypoxia associated with OSA further exacerbate this relationship by altering neuronal activity and reducing white and gray matter in the brain, thereby intensifying depressive symptoms (58). Anxiety is considered another risk factor for OSA occurrence, this is because anxiety may influence brain regions, including the ventromedial prefrontal cortex, cingulate, parietal, and insular cortices, as well as the hippocampus and amygdala. These regions regulate respiratory control (59). These results are consistent with those reported by Duan et al., who found a strong association between anxiety and the increased risk of OSA (60). The connection between stress and sleep apnea is mostly indirect and it varies from one person to the other. Our results show no correlation between stress and OSA risk, which may be due to individual differences in how stress is perceived and experienced. This contrasts with findings from other studies, which have reported a stronger association between stress and the risk of OSA (61).

### Limitations and strengths

This study has some limitations. First, it is a cross-sectional study that suggests a possible association where additional research is needed to establish a definitive causal relationship. Second, selection bias existed as individuals aware of using social media platforms and having internet access were able to participate in this study. In addition, the refusal rate cannot be known. Furthermore, the under-diagnosis of OSA in our study may be due to the young age of our participants along with the limited comorbidities at young compared to older ages. We also recognize the potential bias in our analysis group, specifically the higher proportion of women and the relatively young average age of participants. While these demographic characteristics may limit the generalizability of our findings to broader populations, we aim to perform future studies with more diverse and representative samples, including older populations and a more balanced sex distribution, which are essential to confirm and expand upon our findings. Similarly, reporting certain diseases as diabetes mellitus and hypertension could potentially influence the interpretation of our study, as individuals may be asymptomatic leading to undiagnosed hypertension or diabetes, which might not be fully accounted for in the analysis. Finally, the assessment was through a self-reported questionnaire, thus, the information reported may be biased given that the responders might have reported wrong answers based on misunderstanding of some questions. Future studies incorporating the performance of polysomnography and assessment about snoring or sleepiness during driving are still needed to confirm our results as it remains the gold standard for diagnosing OSA. Similarly, home sleep apnea testing can also be considered as a practical alternative for confirming estimates, offering a more accessible option providing data on the presence and severity of OSA.

Our study has certain strengths. This was the first study to evaluate the risk of OSA in a large sample of Lebanese adults residing in different provinces and to correlate this risk with various demographic and clinical characteristics. In addition, the association of OSA with the level of health, anxiety, and distress was assessed utilizing nation-specific validated questionnaires.

## Conclusion

This study showed that most Lebanese adults have low risk for OSA where the risk is strongly associated with nonmodifiable and modifiable factors. Our findings highlight the need for an intervention targeted to reduce the risk of OSA in Lebanese adults. Further future studies are needed to confirm the associations seen in our findings taking into consideration more specific factors and a follow-up period to assess a strong unidirectional relationship. Moreover, future health campaigns should be conducted to increase awareness among the Lebanese population about preventing and slowing the progression of OSA through lifestyle modifications. This is particularly important given the evidence of clinical under recognition, low awareness and low referral rates of OSA within the medical community in Lebanon. Enhanced awareness and early diagnosis can significantly improve outcomes and reduce the societal and individual impacts of OSA.

## Data Availability

The raw data supporting the conclusions of this article will be made available by the authors, without undue reservation.
